# The potential role of peak nasal inspiratory flow to evaluate active sinonasal inflammation and disease severity

**DOI:** 10.1038/s41598-020-69693-6

**Published:** 2020-07-29

**Authors:** José Araújo-Martins, Carlos Brás-Geraldes, Nuno Neuparth

**Affiliations:** 1 Otolaryngology, Hospital de Torres Vedras, Centro Hospitalar do Oeste – Torres Vedras, Rua Dr. Aurélio Ricardo Belo, 2560-364 Torres Vedras, Portugal; 2 Environmental Health Research Group, CHRC/CEDOC, NOVA Medical School, Lisboa, Portugal; 3 Centro de Estatística e Aplicações, Universidade de Lisboa, Lisboa, Portugal; 4 ADM – Instituto Superior de Engenharia de Lisboa, Lisboa, Portugal

**Keywords:** Respiration, Biomarkers, Prognostic markers, Prognostic markers, Inflammation

## Abstract

Although the pathophysiology of nasal polyposis is incompletely understood, rhinologists have seldom studied it with rhinomanometry or peak nasal inspiratory flow (PNIF) due to technical limitations and the perception that polyp size might impair reproducibility and the usefulness of recordings. The objective of this study is to assess how measures of rhinomanometry and PNIF relate to disease activity. Nineteen patients with polyps, 15 patients with chronic sinusitis without polyps and 11 negative controls were evaluated with active anterior rhinomanometry and PNIF. Sinusitis and polyp patients were re-evaluated after medical treatment. Polyp patients had the highest median Lund-Mackay score (14) and a median Johansen score of 1. PNIF and its variation after treatment were also lowest in this group (median 90 L/min before and after treatment; median variation of 0 L/min). Nasal resistance was similar between groups, and only correlated with Johansen score (Spearman = 0.517, p = 0.048) after treatment. Our study suggests that evaluating polyp patients using rhinomanometry and PNIF may provide useful and reproducible data. Several findings considered together suggest that polyp size is not the main determinant of nasal functional changes in these patients, warranting further studies to verify whether PNIF changes reflect sinus inflammation or merely airway obstruction.

## Introduction

Nasal polyposis (NP) is a chronic nasal inflammatory disease and is considered an endotype of chronic rhinosinusitis (CRS)^1–3^. The prevalence of NP is reported to be 1–4% of the general population^1,4,5^, reaching as high as 32% in cadaver studies^6^. The average incidence of symptomatic NP has been estimated to be around 0.86 and 0.39 patients/1,000 individuals per year in male and female patients, respectively^7^. Medical or surgical treatment significantly improve quality of life but surgery may be required in up to 50% of patients^8^ and 8–25% of those may require revision surgery^1,9^. The socio-economical cost of CRS parallels those of diabetes mellitus, chronic obstructive pulmonary disease and coronary artery disease^10^. Poorly controlled CRS is estimated to cost around 2000€ per year, per patient^11^, based on direct health services use, and indirect costs of absenteism and presenteism. The worsening of associated co-morbidities (ex: asthma, sleep apnea, etc.) also represents an economical burden.

Rhinologists agree that research to determine the most effective treatment plan is a priority^12^ and should be encouraged by proper authorities^13^, with emphasis on the principles of precision medicine and patient centered care^3^. These explicitly recommend the use of relevant clinical biomarkers to elaborate personalized treatment plans.

The central role of epithelial cell lesion in the pathogenesis of NP has long been recognized^14^ and mucosal inflammation has been extensively studied and established as the cause rather than the consequence of NP in experimental models^15,16^. However, little was investigated on the effect of nasal ventilation in NP. Consequently, no nasal ventilation variables are considered as possible disease biomarkers in NP. Epithelial metaplasia and ciliary dysfunction were noted with increased local nasal airflow^17^. Known mesenchimal cell mechano-transduction mechanisms^18^ as well as analogies between endothelial cell transmembrane proteins and shear stress lesion with NP nitric oxide and epithelial disaggregation mechanisms suggest that ventilation could have a role in NP pathogenesis. Recently, computerized fluid dynamic models reinforced this idea by demonstrating increased positive pressure areas where polyps were found^19^. In short, several studies suggest that nasal ventilation may indeed influence the behaviour of NP.

There are several techniques to objectively evaluate nasal ventilation, of which rhinomanometry and peak nasal inspiratory flow (PNIF) are the best studied. Several guidelines, panel reports and consensus help researchers and clinicians to apply these techniques in a standardized and comparable manner^20–22^. A recognized limitation is the inability to obtain measures in a setting of severe obstruction, and polyps may also limit the reliability of results because of reduced reproducibility due to variable size between individuals and throughout time, related to disease behaviour or treatment effect, as reported previously^23^. The future in nasal functional evaluation is moving towards the use of computerized analysis of nasal anatomy based on fluid dynamics models^19,24^, or, possibly new methods for non-invasive evaluations such as those based on thermistors^25,26^, that have been adapted from other areas. Such methods may provide very refined analysis of the patients’ breathing function without the need for artificial ventilation patterns or patient compliance. However, this will also come at the cost of equipment, time, dedicated personnel and specific training to do so. Moreover, they still lack clinical implementation and validation. PNIF, on the other hand, is a well-established method to evaluate nasal function objectively that can be performed by the otolaryngology surgeon during any consultation. This will bear minimal costs and the results will be available immediately. For these reasons PNIF may prove to be an indispensable tool in rhinology practice, as it has been on clinical research^27^.

In summary, NP is a socially relevant disease and new disease biomarkers must be identified to help manage treatment in a personalized manner. Few studies have reported results from PNIF and/or rhinomanometry in NP patient cohorts^23,28–31^ and, to the best of our knowledge, none have specifically tried to measure the effect of polyp size on those techniques. In light of the previous considerations, our aim is to evaluate if polyp size alters, or not, PNIF and rhinomanometry results, which has implications in the validity of testing those techniques as NP disease biomarkers in the future.

We hypothesize that polyp size does not significantly change the results from nasal functional studies. To test that hypothesis, PNIF and rhinomanometry measurements were compared against polyp size. Additionally, the same measurements were recorded in patients with CRS or without nasal mucosa disease. Comparing those groups against NP patients, will help to answer the question of whether the presence of polyps is inevitably associated with poorer nasal ventilation.

## Methods

### Study design

This is a cross-sectional and prospective study. Three patient groups were considered for recruitment: negative controls (NC), without nasal mucosa disease, positive controls, with CRS (without polyps) and patients with NP. This project respected all national and European regulations and legislation concerning good medical practice, clinical research, bioethics and data protection. The study protocol was submitted to and approved by the Ethics Committees from NOVA Medical School and Centro Hospitalar do Oeste. All patients gave their written and informed consent to participate in the study. Given the exploratory nature of the study and scarce information in the literature, a convenience sample size was decided based on examples of published studies^32,33^. The study was developed in the context of a PhD project and funded by a prize from the NOVARTIS|Excellence in Medicine program and a grant from NOVA Saúde (Universidade Nova de Lisboa Rectorate).

### Setting

The study takes place in an Otolaryngology and Head and Neck Surgery clinic in Hospital de Torres Vedras—Centro Hospitalar do Oeste. All eligible patients who met the inclusion criteria were sequentially enrolled after informed written consent was obtained. The main author was responsible for recruiting and evaluating (clinical, endoscopic, PNIF and rhinomanometry measurements) every study participant.

### Patient recruitment and follow-up

Patients were evaluated and recruited to each group according to the diagnostic criteria published in EP3OS^1^. Those patients who met the following exclusion criteria were not enrolled in the study: patients unable to provide written informed consent,with congenital or pos-traumatic anatomic abnormalities,proven or suspected nasal neoplasia; choanal, antro-choanal or isolated single nasal polyps; isolated maxillary or sphenoid sinusitis. All patients had a baseline evaluation with PNIF and rhinomanometry and were medically treated according to the recommendations in the EP3OS document. Patients with CRS were provided with systemic and nasal topical corticosteroid and long-term chlarithromycin. Patients with NP were provided with the same and also leukotriene antagonists. Patients in the CRS and NP were re-evaluated 5–8 weeks after beginning medical treatment.

### Nasal functional evaluations

Active anterior rhinomanometry was performed using a NR6 manometer from GM Instruments^®^ with the research software (4-phase curve analysis) and PNIF was performed with a Youlten debitometer, according to published consensus recommendations^20,21^. A graphic depiction of the equipment and recording set-ups may be found in these guidelines^21^. A twenty minutes acclimatization period was respected and all evaluations were performed before and after nasal decongestion with topical xylomethazoline spray (Vibrocil Actilong^®^)—a puff in each nostril, followed by another 5 min after, and measurements ten minutes after the second puff. All recordings took place in the same room at an average temperature between 20° and 25° and although complete atmospherical standardization would be ideal, it is not strictly necessary^22^.

### Study variables and analysis

Age, gender and smoking status were registered as base population characteristics. The extension of sinusitis was measured using the Lund-Mackay score from computed tomographic scans of the sinuses (each side and total). Polyp size was recorded considering Johansen’s scale^34^, modified according to Kramer et al.^35^, in order to distinguish large (polyp size 3) from obliterative lesions (polyp size 4): 0—no polyps,1—polyps not reaching the middle turbinate lower border,2—polyp not reaching the inferior turbinate lower border; 3—beyond the inferior turbinate lower border; 4—complete obstruction of the nasal airway. PNIF values were recorded before and after decongestion and the difference in the best of these values, before and after treatment was calculated (ΔPNIF). From the rhinomanometry evaluations we recorded resistance at 150 Pa from the left (Re) and right (Rd) nasal cavities and whole nose (Rt), as well as Broms angle (Ve, Vd and Vt) in both inspiratory and expiratory phases.

A descriptive statistics study was performed for all variables. Continuous variables are presented with the average and standard deviation or median and interquartile range, as appropriate, and categorical variables are presented as proportions. Parametric or non-parametric independent or related samples tests were used with Bonferroni correction, according to whether the distribution of the data in each variable was normal or not. The Wilcoxon test was used to compare measurements before and after decongestion and the Chi-squared or Fisher test were used to compare categorical variables. A p-value < 0.05 was considered statistically significant and nasal cavities were compared independently from the patient (i.e. as if they were “different separate individuals”). Effect sizes for comparisons were calculated and interpreted according to Cohen criteria, as described by Pallant^36^.

### Ethical approval

All procedures performed in this study involving human participants were in accordance with the ethical standards of both institutional research committees (NOVA Medical School and Centro Hospitalar do Oeste) and with the 1964 Helsinki declaration and its later amendments and comparable ethical standards.

### Informed consent

Informed consent was obtained from all individual participants included in the study.

### Human research and informed consent

The study was approved by the institutional review board and namely by the ethics committee from NOVA Medical School and Centro Hospitalar do Oeste. All study participants were volunteers and were only included after providing their free and informed consent by writing.

## Results

### Patient recruitment

**Figure 1:**
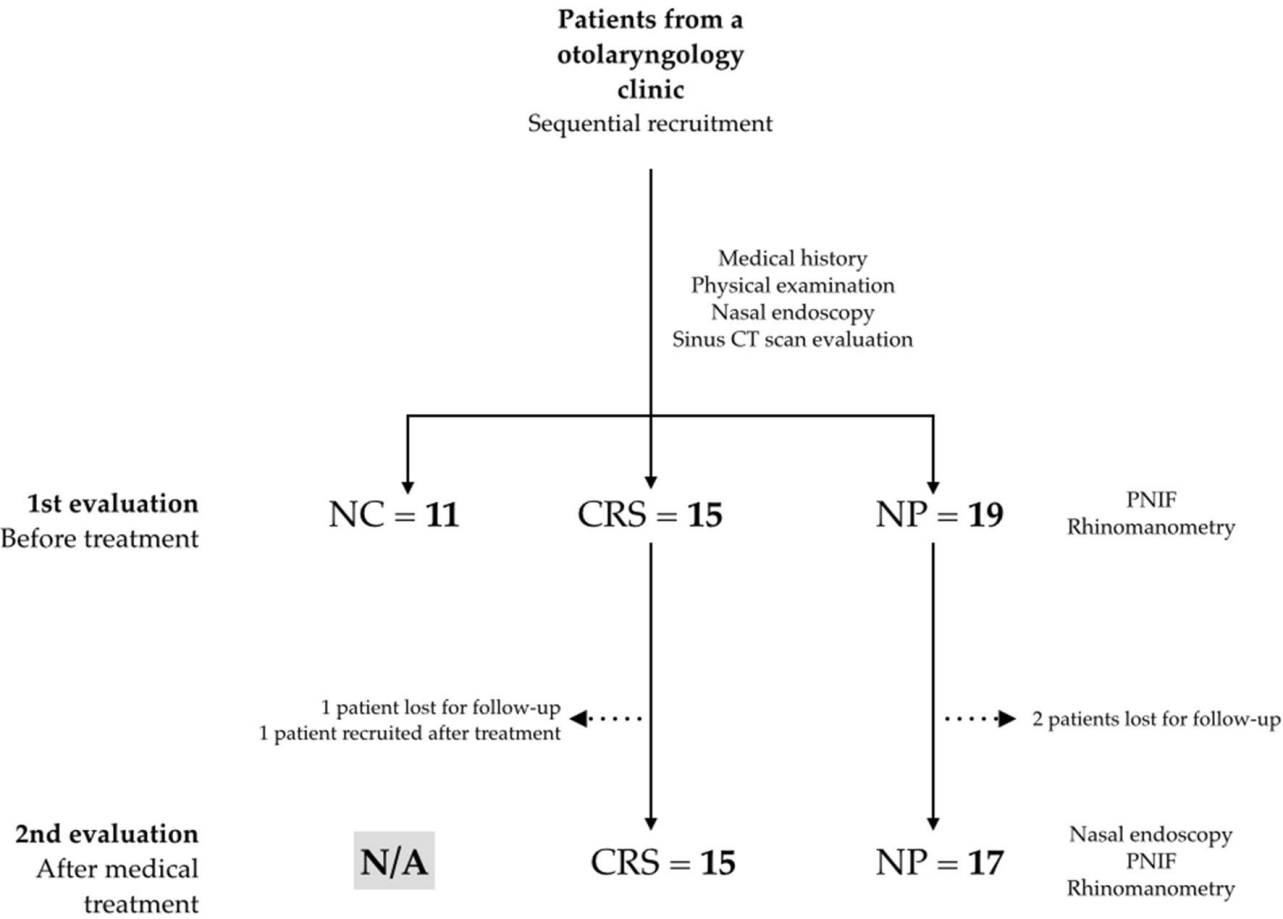
Patient recruitment flowchart. *NC* negative controls, *CRS* chronic rhinosinusitis patients, *NP* nasal polyps patients, *PNIF* peak nasal inspiratory flow, *N/A* not applicable. Patients lost for follow-up failed to show for their scheduled appointments and could not be summoned to return for evaluation. One patient was recruited only after treatment as the first evaluation was before the beginning of the project.

Eleven healthy patients were evaluated, as well as 16 CRS patients (one lost for follow-up after beginning treatment and another recruited only after treatment) and 19 NP patients (two lost for follow-up)—see study flowchart in Fig. 1. When data is missing (due to absent records or inability to measure functional variables), n values for each analysis may differ from N values for each group.

### Population characteristics

The individual descriptive characteristics for all patients can be found in Table 1. Significant differences were found regarding age, smoking status and Lund-Mackay scores. Age difference is significant between groups (p = 0.016) as well as smoking status (p = 0.027), due to the characteristics of the NP group. The proportion of smokers between genders was not significantly different (8/22 females vs 7/24 males, p = 0.755). Lund-Mackay scores are significantly different between groups, although, despite the observed trend for higher values in the NP group, statistical significance could not be reached when comparing CRS to NP patients (p values between 0.07 and 0.17).

**Table 1:** Patient base characteristics.

Group/characteristic	NC (n = 11)	CRS (n = 16)	NP (n = 19)	Statistical diferences between groups
Sex (F/M)	6/5	8/8	8/11	p = 0.274
Age (years)	43 ± 13	46 ± 12	58 ± 12	p = 0.016
Smokers	5 (45%)	8 (50%)	2 (11%)	p = 0.027
Lund-Mackay (right)	0 (0–1)	5.5 (4–6)	7 (5–7.5)	p = 0.000
Lund-Mackay (left)	0 (0–2)	5 (4–6)	7 (6–7)	p = 0.000
Lund-Mackay (total)	0 (0–3)	10 (8–12)	14 (11.5–15)	p = 0.000

Sex is shown as proportion, age as average ± standard deviation, smokers as percentage of smoking patients and Lund-Mackay results as median (P_25_–P_75_).

*NC* negative controls, *CRS* chronic rhinosinusitis patients, *NP* nasal polyps patients, *F/M* female/male.

Statistically significant differences were found in all variables, except sex. See text for inter/intragroup comparisons.

### Polyp size

Average polyp sizes and their changes with treatment are described in Table 2. One patient had bilateral score 4 polyposis in the first evaluation. Three patients showed complete regression of their polyps and an average change of 0.5 in score in each side was observed after treatment, which was significant (right, left and total: p = 0.020, p = 0.023 and p = 0.009, respectively).

**Table 2:** Modified Johansen scale polyp sizes. Results are shown as median (P_25_–P_75_).

Polyp size	Right	Left	Total
Pre-treatment (n = 19)	1 (1–2)	1 (1–2)	3 (2–4)
Post-treatment (n = 17)	1 (0.5–1.5)	1 (0.5–2)	2 (2–2.5)
Statistical differences after treatment	p = 0.020	p = 0.023	p = 0.009
Effect sizes from the differences	r = − 0.400 Medium effect size	r = − 0.389 Medium effect size	r = − 0.528 Large effect size

Three patients showed complete polyp regression after treatment (score result = 0). All post-treatment changes were statistically significant (see text).

### PNIF

A preliminary analysis concluded that there was no significant difference between PNIF values recorded before and after decongestion. With that in mind, we opted to discuss and present baseline results before decongestion, except where noted otherwise (Table 3). The change in PNIF after treatment is shown as ΔPNIF. A significant difference between NC patients and CRS or NP patients was evident and significant (p = 0.010). After treatment, sinusitis and polyp patients showed significantly different average PNIF recordings (p = 0.043). Although other comparisons could not reach statistical significance, the data suggest a clinically significant trend for ΔPNIF to be lower in NP patients (35 L/min in sinusitis vs 0 L/min in polyps). Furthermore, PNIF change after treatment was significant in CRS patients (p = 0.048), but not NP patients (p = 0.674).

**Table 3:** PNIF values (L/min).

PNIF	NC (n = 11)	CRS (n = 16)	NP (n = 19)	Statistical diferences between groups	Effect size from differences between groups
Pre-treatment	150 (110–180) (n = 11)	105 (57.5–125) (n = 15)	90 (65–110) (n = 19)	p = 0.010	r = 1.377 Large effect size
Post-treatment	N/A	120 (100–167.5) (n = 15)	90 (60–120) (n = 17)	p = 0.043	r = 0.727 Large effect size
ΔPNIF	N/A	35 (0–60) (n = 14)	0 (-15–25) (n = 17)	p = 0.057	r = 0.652 Large effect size
Statistical diferences after treatment	N/A	p = 0.048	p = 0.674		
Effect sizes from differences after treatment		r = 0.373 Medium effect size	r = 0.072 Small effect size		

Results are shown as median [P_25_–P_75_]. ΔPNIF values in the CRS group only concern 14 individuals, given losses during follow-up (see Fig. 1).

PNIF shows a trend to be smaller and vary less in NP patients. See text for intergroup comparisons.

*NC* negative controls, *CRS* chronic rhinosinusitis patients, *NP* nasal polyps patients, *N/A* not applicable, *ΔPNIF* change in PNIF after treatment.

### Rhinomanometry

All the recordings from rhinomanometry produced a large volume of data that was submitted to an exploratory analysis. Resistance and flow parameters improved significantly after decongestion in the control group. However, no significant changes were found from the effect of decongestion or the respiratory cycle phase in sinusitis and polyp patients. Also, a multiple variable, mixed-effect model analysis reinforced this impression (unpublished data). To abbreviate and avoid redundancy, rhinomanometry measurements are presented in Table 4 and concern the pre-decongested and inspiratory phase results. Adequate readings were impossible in one CRS patient post-treatment (6.7%) and some patients with large polyps (sizes 3 or 4): 3/19 pre-treatment (15.8%) and 2/17 post-treatment (11.8%).

**Table 4:** Rhinomanometry results—Resistances (Pa cm^−3^ s ) and Broms angles (°).

Rhinomanometry	NC (N = 11)	CRS (n = 16)	NP (n = 19)
**Pre-treatment**			
Right resistance	0.54 (0.47–0.59)	0.38 (0.27–1.13) (n = 15)	0.69 (0.47–1.55) (n = 16)
Left resistance	0.66 (0.46–0.99)	0.64 (0.47–2.34) (n = 15)	0.66 (0.53–1.19) (n = 17)
Total resistance	0.28 (0.23–0.33)	0.26 (0.19–0.45) (n = 15)	0.32 (0.26–0.56) (n = 16)
Right Broms angle	28 (25–30)	21 (15–49) (n = 15)	35 (25–57) (n = 16)
Left Broms angle	34 (25–45)	33 (25–67) (n = 15)	33 (28–50) (n = 17)
Global Broms angle	15 (13–19)	14 (11–24) (n = 15)	18 (14–29) (n = 15)
**Post-treatment**			
Right resistance	N/A	0.60 (0.40–0.94) (n = 15)	0.56 (0.37–0.77) (n = 16)
Left resistance	N/A	0.50 (0.38–0.77) (n = 15)	0.69 (0.30–0.93) (n = 15)
Total resistance	N/A	0.27 (0.17–0.38) (n = 15)	0.26 (0.21–0.32) (n = 15)
Right Broms angle	N/A	31 (22–43) (n = 15)	30 (20–38) (n = 16)
Left Broms angle	N/A	26 (21–38) (n = 15)	35 (17–43) (n = 15)
Global Broms angle	N/A	15 (10–21) (n = 15)	15 (12–18) (n = 15)

Results are shown as median [P_25_–P_75_].

In some cells, n values are lower than the group size because adequate readings could not be obtained from the patient (large polyposis) or because patients were lost for follow-up (see text).

*NC* negative controls, *CRS* chronic rhinosinusitis patients, *NP* nasal polyps patients, *N/A* not applicable, *ΔPNIF* change in PNIF after treatment.

No statistical significant differences or trends were noted and hence p-values are omitted for the sake of clarity (see text).

No statistically significant differences were found between groups and in the same patient after treatment.

### Polyp size vs functional results

In the pre-treatment evaluation, no significant correlation was found in unilateral variables, although Total Lund-Mackay scores did correlate with: global Johansen score—Spearman correlation coefficient (Spearman) = 0.658 (p = 0.003) and PNIF pre-decongestion—Spearman =  − 0.546 (p = 0.000), as well as post-decongestion—Spearman =  − 0.500 (p = 0.001). This didn’t change after excluding NC patients from the analysis.

**Figure 2:**
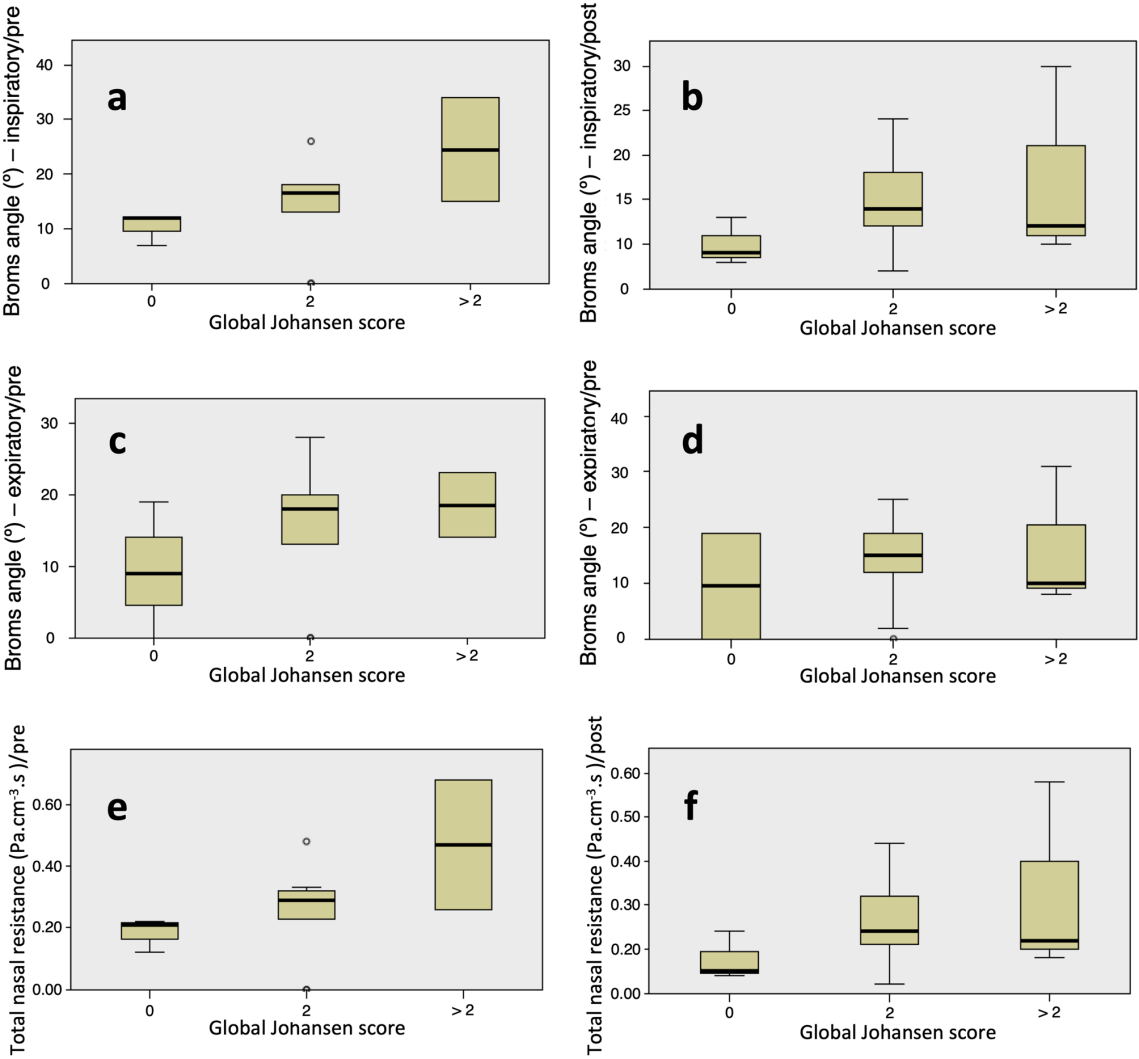
Several graphs illustrate the effect of polyp size on nasal ventilation measures in polyp patients after medical treatment. Broms angles were recorded in ° and nasal resistance as Pa cm^−3^ s. Box-plots are a graphical depiction of the dispersion measures calculated from the recordings obtained from our patients in that given moment and setting—Medians are represented by the thick bars and the lower and upper extremities of the error-bars in the box-plots are P_25_ and P_75_, respectively. In some patients, the values recorded fall outside the calculated P_25_–P_75_ interval and these are represented as outlier circles in (**a**,**c**,**d**,**e**). These outliers stress the point that some patients will show particularly good or bad manometry results despite the presence of small polyps (Global Johansen score of 2). Correlations were mentioned in the text, although no statistical significance is found when comparing different global Johansen score categories. Decongestion may further abate differences from polyp size. Sample sizes are 0 (n = 3), 2 (n = 10) and > 2 (n = 4). (**a**,**c**,**e**) report pre-decongestion recordings, while (**b**,**d**,**f**) report post-decongestion recordings.

In the post-treatment evaluation, however, while unilateral variables didn’t correlate, global Johansen scores correlated with total nasal resistance (Spearman = 0.517, p = 0.048) and inspiratory Broms angle (Spearman = 0.518, p = 0.048), but not expiratory Broms angle (Spearman = 0.303, p = 0.292). PNIF also correlated with rhinomanometry in both pre- and post-decongestion analysis. Pre-decongestion results are: total nasal resistance (Spearman =  − 0.517, p = 0.003), inspiratory Broms angle (Spearman =  − 0.516, p = 0.004) and expiratory Broms angle (Spearman =  − 0.590, p = 0.001). PNIF and Johansen only correlated before decongestion (Spearman =  − 0.49, p = 0.046). All correlations that were found are weak and Fig. 2 illustrates that differences may have reduced clinical significance, especially after decongestion.

## Discussion

Overall, findings from our study suggest that PNIF and rhinomanometry can provide objective measures of nasal ventilation in almost all sinusitis and polyp (except patients with severe size 4 polyps). Published studies have presented PNIF and rhinomanometry values to compare polyp behaviour over time or after treatment^23,28–31^. In our study we have also tried to assess whether polyp size could be a confounding factor that influences those results. When comparing and correlating polyp size against the several measurements and moments, we could only find a few weak correlations with rhinomanometry results. This suggests that differences found with these methods may potentially reflect disease pathophysiology rather than the mechanical effect of polyp size. As such, PNIF and rhinomanometry should definitely be considered in future studies investigating NP pathophysiology. Although sample size may limit statistical power, we found significant differences and relevant effect sizes that are backed up by the apparently consistent and clinically relevant trend in favour of PNIF being able to discriminate patients without polyps from patients with polyps better than rhinomanometry. PNIF should thus be studied as a potential nasal ventilation biomarker of disease severity, particularly considering that it can be easily measured in any otolaryngology outpatient visit.

Below follows a point by point discussion of our work.

The distribution of patient characteristics in our sample is similar to that of others in the literature in the multiple aspects we discuss next. There is a slight male predominance in the NP group, although not the 2:1 reported elsewhere^37^. Studies in caucasian and asian populations agree that polyps tend to present in older individuals^38^, peaking around 50–59 years of age^7^. It has also been reported that in patients with sinusitis, those with polyps tend to be older^39,40^. Our sample shows those same trends. The incidence (but not the gender proportion) of smokers (32.6%) follows that of the Portuguese population^41^. In a manner similar to our patients, other studies have also reported a higher prevalence of smokers in sinusitis groups^42^, as well as a lower proportion of NP patients who smoke, both in national and international cohorts^40,43,44^. Although Lund-Mackay scores aren’t used in clinical decision, they represent the extent of sinus inflammation (even though the score may be different from 0 in any given healthy individual). Increased scores have been associated with more symptoms and the presence of nasal polyps^40,45^ and the same was found in our co-hort. To assess disease severity, 3- or 4-point scales have often been used with good inter-observer consistency in the practice of rhinology^46^. These scales are simple to apply and easy to compare in different studies. Although polyp size may vary over time and does not predict treatment success, patients often tend to present with small polyps regardless of when they present for evaluation^23,29,30^. Our patients also had predominantly small polyps.

With the exception of a score 4 polyp patient, all other individuals in our study could perform a reliable PNIF maneuver, despite the fact that around 7.34% unobstructed caucasians aren’t able to do so^47^. Rhinomanometry at 150 Pa was impossible only in patients with very large polyps (15.8% pre- and 11.8% post-treatment) and also one CRS patient (6.7%). These results demonstrate that these methods can effectively measure nasal ventilation in most patients with NP.

Considering each side of the nose independently in the analysis is important because the same patient may have different polyp sizes. This is possible only because they are functionally separated (no septal perforations). This way, a broader range of nasal airways in different nasal cycle states is also represented in readings before decongestion. Such an analysis effectively doubles sample size in unilateral comparisons (22 nasal cavities in the control group, 32 in sinusitis patients and 38 in polyp patients). We have also considered polyp patients according to global Johansen scores distributed into three categories: 0 represents no polyps, applicable only after successful medical treatment (3 patients); 2 represents small intrameatal polyps (8 individuals before treatment and 10 after treatment); and > 2 represents patients with larger polyps that impinge on the nasal airway corridors reducing their cross-sectional area (11 patients before treatment and 4 patients after treatment). Despite these adjustments, no functional variable correlated with polyp size unilaterally before or after treatment. No statistical differences were found with rhinomanometry (unilateral or global measurements), despite a trend for poorer results in NP patients. While sample size could be the cause, we need to consider the possibility that there is an inherently small magnitude of differences between patients. This may have also compromised unilateral correlations with polyp size. Global variables, however, did show some differences. PNIF in our study appears to be lower and change less after treatment in NP patients, in a magnitude similar to the reports by other authors^23,29,30^. This effect may be independent of polyp size as there were no consistent or relevant correlations with polyp scores. Other studies have reported a correlation of polyp size and PNIF^30^, but that finding was merely a measure of treatment effect. We have analyzed PNIF separately before and after decongestion. Overall differences before and after topical decongestion were not significant, and the comparisons between groups displayed the same significances and followed the same trends. We opted to present pre-decongestion results as they are easier to obtain and compare between studies and also because they represent the natural functional state of nasal function in the patients’ everyday life. PNIF results negatively correlated with Lund-Mackay scores before treatment. Lower PNIF values have been associated with polyps, even when asymptomatic^5^ and an increased probability of requiring treatment over time^23^. In our study, PNIF was similar between sinusitis and polyp patients before treatment. However, significant changes were noted after treatment in CRS patients, but not polyp patients. This happened despite the changes in polyp size mentioned above and in contrast to the absence of significant changes in manometry results. Rhinomanometry results are similar to other reports in the literature^28,31^, which reinforces the idea that there are naturally small differences between groups. Interestingly, if we apply to this and other studies the logarithmic class transformation proposed by Vogt^47^, all groups will be ranked in class 1 for nasal obstruction (less severe), clearly pointing to a difference in the pathophysiology of symptoms in patients with sinonasal inflammation. This may be related to mucosal edema and inflammation induced neuropathic mechanisms^48^. The same mechanism may also possibly explain the negative correlation between Lund-Mackay scores and PNIF that disappears after treatment. The correlation between Lund-Mackay and polyp size, however, is already recognized^49^. Correlations between rhinomanometry and polyp size were only present after treatment and readily disappeared with decongestion. Decongestion has been shown to significantly alter polyp size^50^, and ventilation recordings in patients with allergic rhinitis^51^. We didn’t design our study to answer specifically why decongestion had such a small effect in manometric readings in our sinusitis and polyp patients. It’s hard to find answers in the literature because there are little available studies that discuss such findings in similar populations or study designs. At this time, we can only hypothesize that some resistance to the decongestant effect may be caused by mucus, epithelial barrier or endothelial changes due to the effect of inflammation or tissue remodeling.

Broms method allows calculation of nasal airway resistance at the point where the rhinomanometric curve intersects a circle with radius 200 (crossing both axes at 200 Pa and 200 cm^3^ s^−1^) and the angle from a line that connects such point to the origin of the graph will be lower the higher the resistance in that nasal cavity. Broms angles were presented because they complement or may substitute nasal resistance values when they cannot be measured in a given patient. Broms angle was also the only dimension that we could measure when considering the different phases of respiration—inspiration and expiration. While inspiratory values behaved similarly to nasal resistance values, expiratory values never correlated with polyp size and there were no significant differences in multiple comparisons. Given that airflow patterns in the nasal cavity are different when comparing inspiration to expiration, we must consider the hypothesis that the difference found may represent a baseline pathophysiological difference. We propose that in future studies researchers make an effort to discriminate inspiratory from expiratory pathophysiology.

In conclusion, PNIF and rhinomanometry provide measurable results in NP and polyp size doesn’t appear to significantly influence recordings, especially if pre- and post-decongestion readings are obtained. The results from this study are consistent with other reports in the literature. It appears differences between sinusitis patients with or without polyps may be naturally small and PNIF may be a more useful clinical biomarker of disease severity. Despite the limitations in our study, its results should encourage us and other groups to continue investigating the clinical usefulness of PNIF and rhinomanometry, as well as their contribution to the study of NP pathophysiology.

## Data Availability

The datasets generated during and/or analysed during the current study are available on reasonable request from either the corresponding author or from the Research Center of the host institution (Centro Hospitalar do Oeste) via e-mail: investigacao@choeste.min-saude.pt.
